# Increasing Performance of Spiral-Wound Modules (SWMs) by Improving Stability against Axial Pressure Drop and Utilising Pulsed Flow

**DOI:** 10.3390/membranes13090791

**Published:** 2023-09-12

**Authors:** Christian Kürzl, Martin Hartinger, Patrick Ong, Roland Schopf, Simon Schiffer, Ulrich Kulozik

**Affiliations:** 1 Food and Bioprocess Engineering, TUM School of Life Sciences, Technical University of Munich, Weihenstephaner Berg 1, 85354 Freising, Germany; 2 Food Process Engineering, TUM School of Life Sciences, Technical University of Munich, Weihenstephaner Berg 1, 85354 Freising, Germany

**Keywords:** pulsed flow, module stability, axial pressure loss, telescoping, membrane performance

## Abstract

Spacer-induced flow shadows and limited mechanical stability due to module construction and geometry are the main obstacles to improving the filtration performance and cleanability of microfiltration spiral-wound membranes (SWMs), applied to milk protein fractionation in this study. The goal of this study was first to improve filtration performance and cleanability by utilising pulsed flow in a modified pilot-scale filtration plant. The second goal was to enhance membrane stability against module deformation by flow-induced friction in the axial direction (“membrane telescoping”). This was accomplished by stabilising membrane layers, including spacers, at the membrane inlet by glue connections. Pulsed flow characteristics similar to those reported in previous lab-scale studies could be achieved by establishing an on/off bypass around the membrane module, thus enabling a high-frequency flow variation. Pulsed flow significantly increased filtration performance (target protein mass flow into the permeate increased by 26%) and cleaning success (protein removal increased by 28%). Furthermore, adding feed-side glue connections increased the mechanical membrane stability in terms of allowed volume throughput by ≥100% compared to unmodified modules, thus allowing operation with higher axial pressure drops, flow velocities and pulsation amplitudes.

## 1. Introduction

The membrane-based separation of a feed solution into the permeable components (permeate) and the retained components (retentate) is widely applied across various industries. Within this process, the main challenge is controlling deposit formation, i.e., fouling, which results from the accumulation of retained feed components on and in the membrane structure [1,2,3,4,5]. With deposits acting as a secondary selective layer, this causes separation efficiency in terms of flux and protein permeation decreasing gradually during filtration [6,7,8]. Additionally, frequent cleaning cycles are required to maintain membrane performance and product quality due to the feed- and temperature-dependent occurrence and progression of biofouling [9,10,11,12,13]. For dairy applications, one important example is the fractionation of skim milk protein into its major protein components: whey proteins (particle diameter d_P_ = 3–6 nm [14]) and casein micelles (d_P_ = 20–300 nm [14]) via microfiltration (MF; nominal pore size = 0.1–0.3 µm). This poses a particular challenge for the application of fouling and cleaning as casein micelles can form highly compressible and cross-linked gel layers at high concentrations and high-pressure conditions [15,16,17,18,19]. Deposit formation cannot be reverted by pressure release [20], and accumulated protein can only be incompletely removed by rinsing steps [1,2,21].

Besides feed composition, the membrane performance largely depends on processing conditions. Up to limiting conditions, transmembrane pressure (TMP) increases can be used to achieve gains in flux. However, beyond limiting conditions, further increases in TMP do not cause further flux increases but solely result in additional, partly irreversible [20] deposit compaction and fouling [7]. Another option to enhance membrane performance is by increasing the flow velocity v, and thus the wall shear stress τ_w_, as this reduces fouling [22,23,24,25,26]. Nonetheless, the maximum applicable crossflow velocities in SWMs are limited by geometry-related or constructional aspects, thus also limiting fouling control and cleanability.

Regarding membrane geometry, fouling depends on the membrane length and varies along the membrane [27]. Due to friction, an axial pressure loss (Δp_L_) over the membrane length is induced. This causes a decrease in TMP and thus in the fouling intensity from the module inlet towards the module outlet [27]. The main industrially used module configurations are ceramic tubular membranes (CTMs), hollow-fibre membranes (HFMs) and SWMs, which all have their typical pros and cons. Compared to CTMs and HFMs, SWMs offer the highest packing density, i.e., active membrane area per module, and thus the highest whey protein mass flow per module [28]. On the contrary, SWMs suffer from flow shadows behind spacer filaments [29,30,31,32,33,34,35,36,37] and, therefore, limited cleanability [29] and mechanical stability. In SWMs, the membrane permeate pockets are formed by glueing together individual membrane sheets, which are then wrapped around a central permeate collection tube and fixed by an outer hull. Thus, the SWMs’ stability mainly depends on the stability of glued bond joints and the friction between the membrane sheets, which results from the strength of the wrapping.

The stability of bond joints depends on several construction-related aspects, such as the glue composition [38], the design [39] and overlapping length of connections [40] and the glue layer thickness [38]. Apart from that, process-related aspects, such as the speed of stress application, the intensity and kind of stress [38], the process temperature [38] and the duration of stress application, have significant effects [38,41]. Due to membrane pockets formed by glueing together membrane sheets, the permeate side is susceptible to failure, especially by negative TMP, which stresses bond joints via peeling and can cause tearing of the membrane pockets. To avoid this, manufacturers usually limit the maximum negative TMP to around 0.3 bar.

A more common failure mechanism in SWMs is telescoping, which describes an axial displacement of the membrane pockets caused by frictional losses along the module (∆p_L_) acting on the membrane envelope. Hence, the strength of the wrapping determines the amount of friction between membrane sheets and thus its stability against axial displacement. However, besides a lower risk of telescoping, stronger wrapping can also press spacers into the membrane surface, thus reducing the active membrane area and even disrupting the selective layer [42]. The trade-off between stability against axial deformation and membrane performance led to manufacturers limiting the friction-related axial pressure drop ∆p_L_ to 1.3 bar m^−1^ despite the commonly added stability support against axial displacement via anti-telescoping devices (ATD). With ∆p_L_ and thus v being limited, this significantly restricts options to control deposit formation, e.g., by conventionally established higher crossflow velocities in SWMs compared to the other module types described above.

Several process-oriented approaches trying to increase membrane performance have been investigated to cope with this limitation. One example is applying pulsed flow, i.e., a non-steady flow defined by its amplitude, in other words the difference between maximum and minimum flow and pressure conditions, and its frequency. Several studies demonstrated the positive influence of pulsed flow on filtration [31,43] and cleaning performance [29,44] for various feed solutions, including milk. For membranes containing spacers, such as SWMs, particularly strong effects of pulsed flow on filtration and cleaning performance were reported due to pulsed flow reducing flow shadows due to enhanced turbulence [29,31] at high frequencies [44,45,46,47,48,49] and amplitudes [44,45,46,47,49].

However, some aspects exacerbate the transferability of lab-scale results to industrial-scale SWM modules. Firstly, the approaches to pulsation creation used in previous lab-scale studies either included piston or bellows units [45,50] or specialised inductively controlled pumps that could create pulsed flow by rapidly increasing and decreasing pump capacity [29,31,44]. The former approach temporarily induced distinct back-pressure and is thus incompatible with SWMs. To the authors’ knowledge, the latter one is unavailable on a larger scale. Secondly, the transferability of results from studies with FSMs to SWMs has been considered to be problematic for certain spacer geometries due to the curvature of the feed channel and its influence on the radial distribution of v [37]. Thirdly, due to the limited applicable pressure drops in SWMs, the highest applicable flow velocity and pulsation amplitude are also limited. In particular, positive results for pulsed filtration in a previous study using an SWM-like flat sheet membrane system (FSM) were found for pressure losses up to 2.55 bar m^−1^ [31], which is beyond the allowed limit of SWM modules. Thus, the advantage of pulsed flow might be reduced or absent for current SWM modules.

Hence, the potential beneficial effect of pulsed flow in filtration and cleaning remains to be evaluated for industrial-scale SWMs. Therefore, a novel approach was developed to create pulsed flow without back-pressure from the permeate side or relying on rapid pump capacity ramps. Then, pulsed flow can be utilised to assess the efficacy of pulsed flow MF of skim milk and subsequent membrane cleaning in SWMs. With previous studies observing improved pulsed flow efficiency for increased amplitudes and at pressure drops above the current limits of industrial SWMs, stability-enhanced modules could support the efficiency of pulsed flow manifold by enabling higher crossflow velocities, axial pressure drops and pulsed flow amplitudes. Hence, this study also investigates an approach to improve module stability against telescoping by adding glue connections on the feed side between membrane pockets, as this should provide additional resistance against the displacement of individual sheets in the axial direction.

Accordingly, this study aims to overcome the limitations of SWMs by two means. The first one is process-oriented and functions by modifying an existing plant for utilising pulsed flow and then assessing its efficacy in filtration and cleaning. The second approach is membrane-oriented and functions by creating a more robust SWM by adding glue connections on the feed side between membrane pockets, including spacers, to enhance its mechanical stability. For comparing steady and pulsed flow, filtration performance was evaluated in terms of permeate flux, protein permeation and protein mass flow. Cleaning success was evaluated hydrodynamically by measuring the flux recovery ratio (FRR) and chemically by analysing the protein removal achieved during cleaning. To investigate the effect of additional glue connections on module stability, the axial displacement of membrane layers in an unmodified and a glued membrane system was measured at different flow rates and radial distances to the module centre.

## 2. Materials and Methods

### 2.1. Filtration Plant and Experimental Design

**Figure 1:**
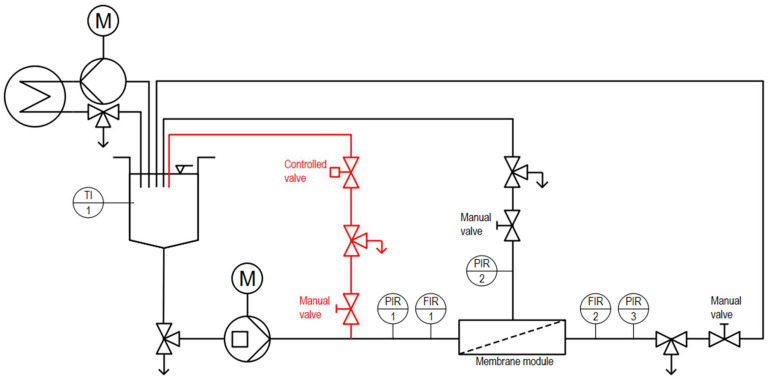
Piping and instrumentation (P and I) diagram of an established membrane filtration plant setup (black parts) consisting of feed pump, feed tank, sampling valves, manual throttling valves, a heating cycle consisting of another feed pump and heat exchanger, as well as various flow, pressure and temperature sensors. The red parts show the complimentary addition of a controlled bypass for utilising pulsed flow, including a manual throttling valve to control the pulsation amplitude and a controlled valve to control the pulsation frequency.

An established pilot-scale filtration plant (Figure 1) was designed to resemble a typical industrial setup. It mainly consisted of a double-screw-type displacement pump (FDS 2-3, Fristam Pumpen KG, Hamburg-Bergedorf, Germany), which is insensitive to moderate pressure surges and commonly used in several dairy applications in which, e.g., highly viscous fluids such as milk concentrates need to be processed, and a membrane housing with the established module configuration 6338 (length L = 0.96 m; diameter d = 0.16 m). Additionally, pressure sensors and flow meters allow the monitoring and controlling of the transmembrane pressure TMP (see Equation (1)), feed flow rate and permeate flux J (see Equation (2)).

(1) $$TMP=\frac{p_{1}+p_{2}}{2}-p_{3}$$

where p_1_ is the feed-side pressure, p_2_ is the retentate-side pressure and p_3_ is the permeate-side pressure.

(2) $$J=\frac{{\dot{V}}_{per}}{A_{membrane}}$$

where ${\dot{V}}_{\mathrm{per}}$ is the permeate flow rate and A_membrane_ is the membrane area.

A separate heat exchanger loop combined with a temperature sensor enables precise temperature control of the filtration fluids before entering the membrane loop. Thus, the system can process various filtration feeds at defined temperatures, withstanding pressure surges and varying the installed membrane module’s geometry and pore size.

#### 2.1.1. Plant Modification and Experimental Design to Utilise Pulsed Flow

To enable applying a pulsed flow to a standard membrane filtration plant setup without a pump capable of rapidly transitioning between high and low flow rates, a controlled bypass was added upstream of the membrane inlet flow meter and pressure sensor (see Figure 1). Accordingly, the bypass-related flow rate or pressure reductions could be monitored with installed sensors. The bypass comprised a relay-controlled pneumatic valve capable of fully closing or opening the bypass within 0.5 s at defined intervals. Hence, by determining the phase durations where the bypass was open (Δt_min_) or closed (Δt_max_), flow rate and pressure reach their minimum (${\dot{V}}_{\min}$, v_min_ and TMP_min_) or maximum (${\dot{V}}_{max}$, v_max_ and TMP_max_), respectively, and thus control the pulsation frequency f (Equation (3)).

(3) $$f=\frac{1}{{\Delta t}_{max}+{\Delta t}_{min}}$$

The additional manual valve allows control over the extent of flow rate reduction when the bypass is opened and thus the amplitude of pulsed flow in terms of flow rate (Equation (4)), flow velocity (Equation (5)) and TMP (Equation (6)).

(4) $$\Delta \dot{V}={\dot{V}}_{max}-{\dot{V}}_{min}$$

(5) $$\Delta v=v_{max}-v_{min}$$

(6) $${\Delta TMP}_{cycle}={TMP}_{max}-{TMP}_{min}$$

where Δ$\dot{V}$ is the amplitude of flow rate, Δv is the amplitude of flow velocity and ΔTMP_cycle_ is the amplitude of TMP. It is to be noted that the flow velocity was calculated for a theoretical channel height without a spacer and a channel width of the SWM’s spiral length. Hence, calculating *v* for spacer-filled channels can only provide a rough estimation, with local values highly depending on the position relative to the spacer grid.

All pulsed flow experiments were conducted at 50 °C, resembling a typical industrial filtration temperature [9]. The membrane was an MF SWM (GE JX6338C50) with a nominal pore size of 0.3 µm, the material polysulfone, a spacer height of 1.27 mm (50 mils), an active membrane area of 15.6 m^2^, a diameter of 6.3 inches (16 cm) and a length of 38 inches (96 cm). Pasteurised skim milk (74 °C, 28 s) from a local dairy (Molkerei Weihenstephan, Freising, Germany) was used for deposit formation in all steady and pulsed flow filtration and cleaning experiments. Apart from filtration, deionised (DI) water was used in all other steps, either pure for rinsing or combined with chemicals for cleaning. As high frequencies [45,46,47,48,49] and amplitudes [45,46,47,51] were found to be beneficial for pulsed flow efficiency, the respective maximum values that were possible with the current setup were used in the pulsed flow filtration and cleaning experiments. Regarding TMP, the average values were chosen to resemble typical process conditions. TMP_avg_ during cleaning resembles the lowest possible value without reaching negative values for TMP_min_ and still enabling the identical flow velocity amplitude as during filtration (details see below).

##### Filtration Experiments

Before filtration, the membrane was conditioned with Ultrasil 69 (0.4% *v*/*v*, Ecolab Deutschland, Monheim am Rhein, Germany) at 50 °C for 20 min. After an intermediate rinsing step to avoid chemical residues, milk was heated to the process temperature by the heat exchanger loop, and filtration was initiated. Pulsed flow filtration was conducted with f = 0.5 Hz, the highest technically possible ∆$\dot{V}$ = 10 m^3^ h^−1^, due to pump capacity limitations (${\dot{V}}_{\max}$ = 14 m^3^ h^−1^ with Δp_L, max_ = 0.83 bar m^−1^ and v_max_ = 0.37 m s^−1^, ${\dot{V}}_{\min}$ = 4 m^3^ h^−1^ with Δp_L, min_ = 0.14 bar m^−1^ and v_min_ = 0.11 m s^−1^, $\dot{V}$_avg_ = 9 m^3^ h^−1^ with Δp_L, avg_ = 0.35 bar m^−1^ and v_avg_ = 0.24 m s^−1^) and ΔTMP_cycle_ = 1.50 bar (TMP_max_ = 1.75 bar, TMP_min_ = 0.25 bar, TMP_avg_ = 1.00 bar). The average TMP_avg_ and $\dot{V}$_avg_ were used for conducting comparative steady flow filtration runs. During the filtration duration of 60 min, samples were taken from permeate and retentate sample ports after 5, 10, 15, 30, 45 and 60 min. Protein permeation for a specific milk protein P_i_ was calculated by Equation (7) by its respective concentrations in the permeate c_i, p_ and retentate c_i, r_. Similarly, c_i, p_ and Flux *J* were used to calculate an individual protein’s permeating mass flow ${\dot{m}}_{i}$ into the filtrate (Equation (8)).

(7) $$P_{i}=\frac{c_{i,\;p}}{c_{i,\;r}}$$

(8) $${\dot{m}}_{i}=J\cdot c_{i,\;p}$$

After each filtration experiment, the membrane was rinsed and then cleaned with combined caustic (0.8% *v*/*v*, Ecolab Germany) and enzymatic (0.3% *v*/*v* Ultrasil 67, Ecolab GmbH, Monheim am Rhein, Germany) cleaning agents for 40 min, followed by another rinsing step and an acidic cleaning step (0.4% *v*/*v* Ultrasil 75, Ecolab GmbH, Monheim am Rhein, Germany) for 20 min at 50 °C. To verify sufficient cleaning success and thus ensure long-term membrane functionality, the membrane’s pure water flux was measured before each filtration run.

##### Cleaning Experiments

Before filtration, the membrane was conditioned, and the initial water flux J_0_ was measured. Filtration was then conducted for 40 min at 50 °C, $\dot{V}$ = 5 m^3^ h^−1^ and TMP = 1.7 bar with skim milk. Afterwards, the milk was drained, and the membrane system was carefully rinsed to remove bulk milk and loosely bound material. The following cleaning experiments were conducted with NaOH at pH 11.3 (c_NaOH_ = 0.03%) for 20 min at 50 °C in circulation under either steady or pulsed flow mode. Due to the NaOH solution volume being high compared to the membrane area to be cleaned (yielding a specific cleaning volume of 6.4 L per square meter of active membrane area), an excess of cleaning agent compared to the amount of protein to be removed was present. Thus, the experiments should not be affected by the excessive consumption of cleaning agents causing distorted protein removal or changes in the pH. Pulsed flow cleaning was conducted with f = 0.5 Hz, the maximum technically viable ∆$\dot{V}$ = 10 m^3^ h^−1^ (${\dot{V}}_{\max}$ = 14 m^3^ h^−1^ with Δp_L, max_ = 0.83 bar m^−1^ and v_max_ = 0.37 m s^−1^, ${\dot{V}}_{\min}$ = 4 m^3^ h^−1^ with Δp_L, min_ = 0.14 bar m^−1^ and v_min_ = 0.11 m s^−1^, $\dot{V}$_avg_ = 9 m^3^ h^−1^ with Δp_L, avg_ = 0.35 bar m^−1^ and v_avg_ = 0.24 m s^−1^) and ∆TMP_cycle_ = 1.00 bar (TMP_max_ = 1.15 bar, TMP_min_ = 0.15 bar, TMP_avg_ = 0.60 bar). The average TMP_avg_ and $\dot{V}$_avg_ were used for comparative steady flow cleaning runs. For evaluating chemical cleaning success in terms of protein removal, samples were taken from the feed vessel after 20 min cleaning. Subsequently, the cleaning solution was drained, the system rinsed, and the water flux after cleaning J_1_ was measured to evaluate the hydraulic cleanliness in terms of flux recovery ratio (FRR) (see Equation (9)) reached by the applied cleaning protocol. If the cleaning evaluation indicated incomplete cleaning (FRR < 90%), an additional cleaning procedure with industrial cleaning agents, analogous to filtration experiments, was conducted to evaluate long-term membrane functionality.

(9) $$FRR=\frac{J_{1}}{J_{0}}$$

It is to be noted that while identical pulsation frequencies as in previous FSM studies could be achieved with this approach and setup, the maximum applicable amplitudes and flow velocities were significantly lower in the current study due to limitations in pump capacity.

#### 2.1.2. Membrane Modification and Experimental Design to Investigate Increased Axial Pressure Drops

To investigate increased axial pressure drops, the plant’s double-screw-type displacement pump (see Figure 1) was replaced by a larger centrifugal pump capable of creating a feed pressure of 4.8 bar and a maximum feed flow rate of 45 m^3^ h^−1^. In this scenario, experiments were conducted with used membranes put out of operation at an industrial plant to be free for establishing potentially destructive conditions. The membranes were provided by a local dairy, where they had been used for the filtration of dairy fluids for several months. The membranes (Koch Industries, Wichita, KS, USA) had a separation range of 10 kDa, a 31 mil (0.79 mm) diamond-shaped spacer and an active membrane area of 19.1 m^2^, a diameter of 6.3 inch (16 cm) and a length of 38 inch (96 cm). It is to be noted that the used membranes showed no apparent membrane failures despite a few areas with dislocated spacers between non-displaced membrane sheets.

**Figure 2:**
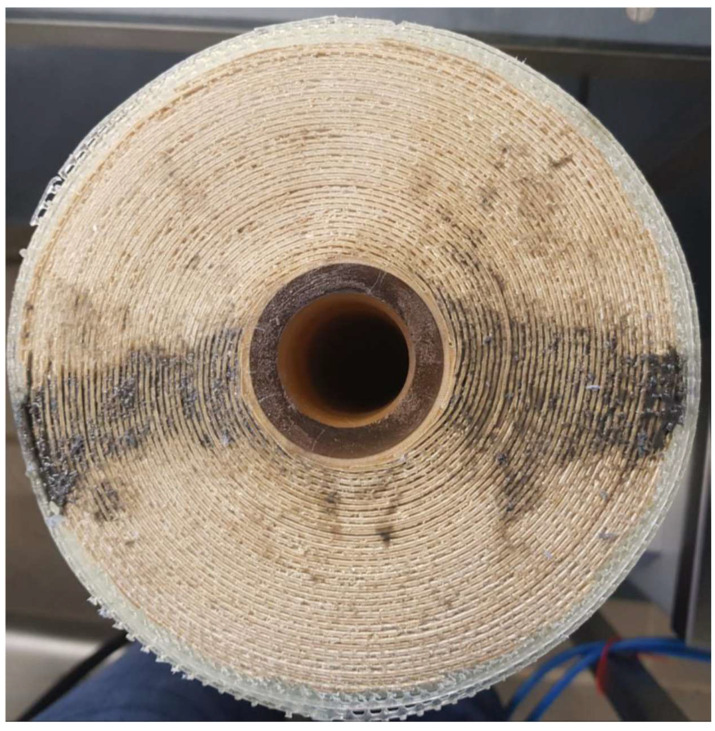
The membrane module was modified by adding glue dots (see black areas) radially along the membrane diameter.

To assess the effect of feed-side glue connections on membrane stability, modified membranes were obtained by inserting a two-component adhesive (Araldite 2014-1, Huntsman Corporation, Salt Lake City, UT, USA) into the dry spacer channels with a syringe and thus glueing together the membrane sheets. This procedure resulted in semi-circular glue connections (d = 2 cm) placed in a radial direction along the membrane diameter (Figure 2). After hardening for several days, the modified membranes were comparatively assessed with unmodified membranes for their axial pressure drop stability.

For axial stability experiments, the membranes were initially rinsed with deionised (DI) water with open permeate valves to allow the permeate pockets to be filled. To simulate the filtration of fouling-intensive feeds such as skim milk, where permeate production is substantially low and thus the influence of flux on the length dependency of crossflow velocity is negligibly small, axial stability experiments with water were conducted with the permeate valve closed (TMP = 0.0 bar). The membrane was then subjected to an initial axial pressure drop of 0.3 bar m^−1^ for 5 min. After assessing the axial displacement relative to the permeate collection tube at four equidistant points (radial distances 3.3 cm, 4.5 cm, 5.8 cm, 7.0 cm) in the radial direction of the SWM with a Vernier calliper, this procedure was repeated, increasing the axial pressure drop by 0.2 bar m^−1^ up to 1.5 bar m^−1^. This approach allowed the evaluation of the displacement depending both on the applied axial pressure drop and the radial distance of displaced membrane sheets to the permeate collection tube.

**Figure 3:**
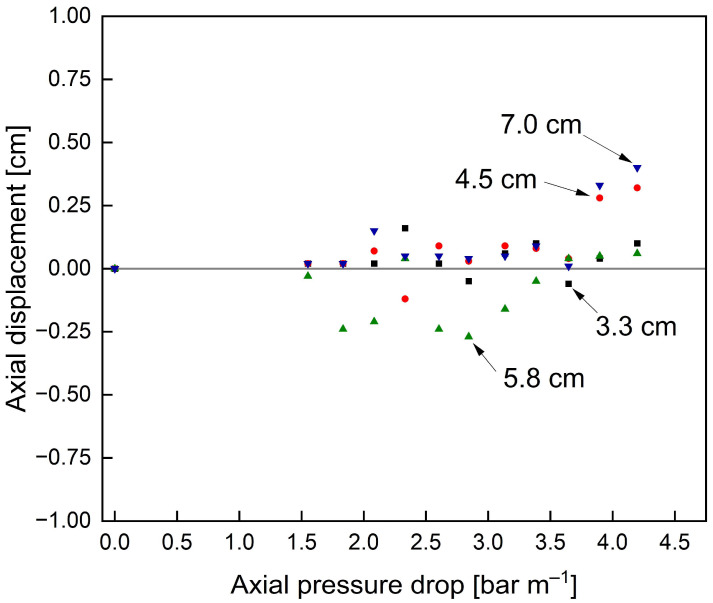
Influence of the axial pressure drop and radial position on the axial displacement of membrane sheets with an ATD. The grey reference line indicates no axial displacement.

Preliminary experiments with unmodified membranes and an ATD showed no significant displacement at either radial position for pressure drops < 4.0 bar m^−1^ (see Figure 3), contrary to industrial reports and restrictions stated by membrane manufacturers. This contradicting observation is presumably due to displacements with ATD only caused by long-term stress, as bond joints and polymers are known to migrate under constant stress [41]. As these long-term scenarios are hard to reproduce at lab scale, the following experiments were conducted without an ATD to exclusively assess the axial stability of the membrane module without the support of an ATD.

### 2.2. Chemical and Statistical Analyses

The contents of caseins and whey proteins in filtration and cleaning samples were analysed by reversed-phase high-performance liquid chromatography (RP-HPLC) as described by Dumpler et al. [52]. Agilent ChemStation software (Rev. B.04.03) was used to analyse RP-HPLC chromatograms.

Data were plotted, fitted and statistically evaluated using OriginPro 2021 (OriginLab Corporation, Northampton, MA, USA). Statistical significance between data sets was assessed using a one-way analysis of variance (ANOVA) at the 5% level (*p* < 0.05). Depicted error bars represent the standard deviation of replicates, whereas all cleaning and filtration experiments were conducted at least in triplicates. Due to membrane failure/rupture accompanying axial displacements, stability experiments could only be conducted as single runs.

## 3. Results and Discussion

### 3.1. Optimisation of SWM’s Process Efficiency via the Utilisation of Pulsed Flow

#### 3.1.1. Validation of Plant Modifications

**Figure 4:**
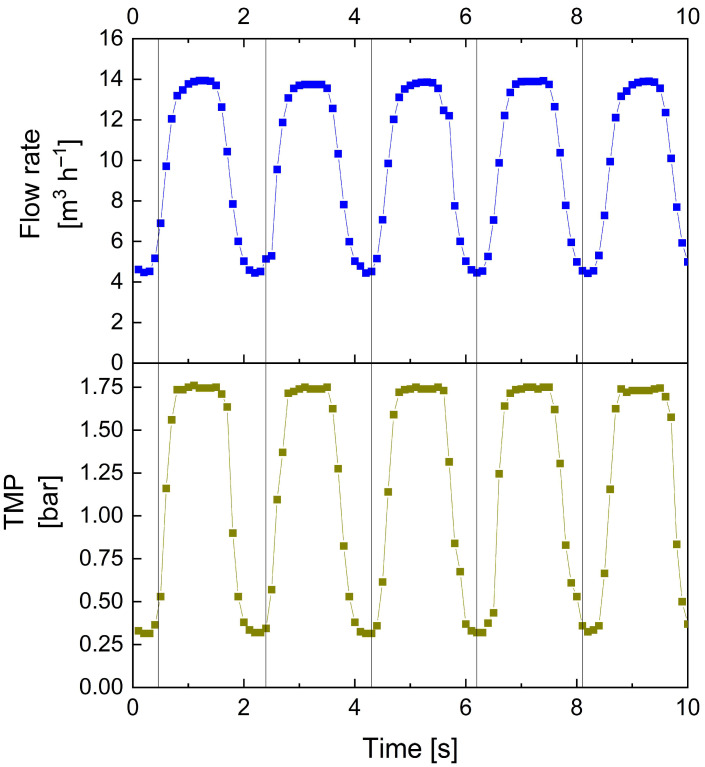
Time-resolved progression of feed flow rate and TMP over several flow cycles during pulsed filtration. Pulsed flow conditions: Δ$\dot{V}$ = 10 m^3^ h^−1^ (${\dot{V}}_{\max}$ = 14 m^3^ h^−1^, ${\dot{V}}_{\min}$ = 4 m^3^ h^−1^, $\dot{V}$_avg_ = 9 m^3^ h^−1^) and ΔTMP_cycle_ = 1.50 bar (TMP_max_ = 1.75 bar, TMP_min_ = 0.25 bar, TMP_avg_ = 1.00 bar).

As larger pumps are normally incapable of rapidly producing quickly transitioning conditions between high and low flow rates, pulsed flow was created by installing a bypass, temporarily reducing the pressure and flow rates reaching the membrane module. An overview of the resulting pulsed flow characteristics is given in Figure 4.

Figure 4 depicts the time-resolved progression of flow rate $\dot{V}$ and TMP. With this approach to pulsation creation and the specific setup used in this study, a maximum frequency of 0.5 Hz with an amplitude >10 m^3^ h^−1^ could be realised. Hence, compared to previous lab-scale studies using steep transitioning ramps of inductively controlled pumps to generate pulsed flow [29,31,44,53], the same maximum frequencies can be achieved at tenfold higher flow rates. Also, the profiles of flow rate and TMP progression correspond to those of lab-scale experiments with rapid ramps creating pulsed flow [44]. It is to be noted that while other valves with shorter opening and closing times might enable higher pulsation frequencies, they might also induce intensified pressure surges on the plant equipment, which could cause enhanced wear. However, the current setup with f = 0.5 Hz did not cause any damage or wear on sensitive plant components, such as sensors or valves, within a pulsed flow operation of four months. Contrary to other approaches of creating pulsed flow, such as via bellows or piston units [45,50], it is also of advantage that the occurrence of negative TMP values can be avoided. This is particularly important for SWMs, where negative TMP must not exceed 0.3 bar, according to membrane manufacturers´ specifications, as this would stress the bond joints of membrane pockets and could result in membrane failure. Overall, the bypass as a technical option to produce pulsed flow conditions resembles a low-effort and cost-efficient approach to creating similar pulsed flow profiles on a pilot scale with industrially sized membranes as in lab-scale studies. Nonetheless, it is to be noted that with this novel approach to pulsation creation, the energy efficiency is decreased compared to that of the previous system using rapid pump capacity ramps [31,44]. In this case, pumps will not alternately increase and decrease in pump capacity, but are instead continuously run at maximum capacity, despite a large share of flow temporarily not reaching the membrane during the low-flow pulsation phases.

#### 3.1.2. Influence of Pulsed Flow on Filtration Performance in Industrial-Scale SWMs

**Figure 5:**
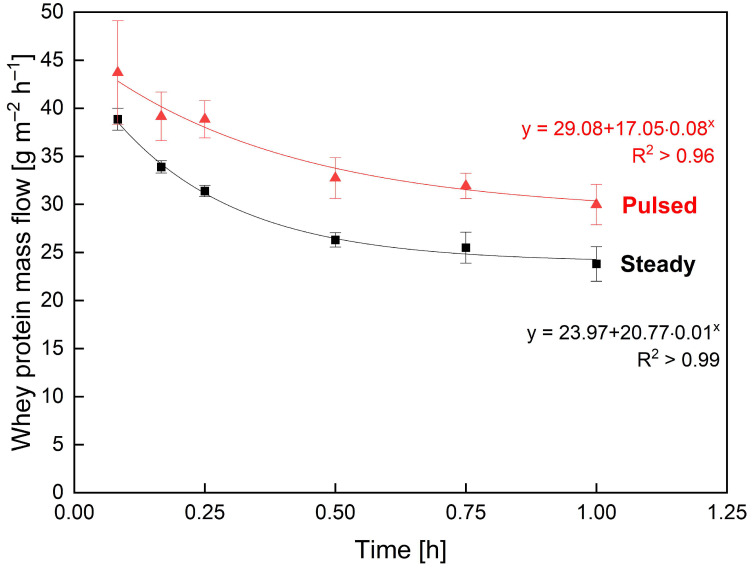
Influence of steady (black squares) and pulsed (red triangles) flow on whey protein mass flow during skim milk MF. Filtration conditions Δ$\dot{V}$ = 10 m^3^ h^−1^ (${\dot{V}}_{\max}$ = 14 m^3^ h^−1^, ${\dot{V}}_{\min}$ = 4 m^3^ h^−1^, $\dot{V}$_avg_ = 9 m^3^ h^−1^) and ΔTMP_cycle_ = 1.50 bar (TMP_max_ = 1.75 bar, TMP_min_ = 0.25 bar, TMP_avg_ = 1.00 bar).

The mass flow resulting from flux and permeation was analysed to assess the effect of pulsed flow on the time-resolved filtration performance in industrial SWMs during 60 min filtration. The strongest impacts of pulsed flow were reported for high frequencies [44,45,46,47,48,49] and amplitudes [44,45,46,47,49]. Accordingly, pulsed flow experiments were run with the best combination of frequency and amplitude applicable to the current setup. The mass flow of whey protein, i.e., the targeted permeating component (Figure 5), for pulsed flow was permanently increased over that of steady flow throughout filtration. While the whey protein mass flow with steady flow decreased from 38.9 g m^−2^ h^−1^ by 39% to 23.8 g m^−2^ h^−1^, pulsed flow decreased from 43.7 g m^−2^ h^−1^ by 31% to 30.0 g m^−2^ h^−1^. Hence, the initial mass flow (+12%), steady-state mass flow (+26%) and its decrease during filtration (−21%) were all improved with pulsed flow. These results demonstrate an improved initial and continuous deposit control with pulsed flow resulting in a 26% increased whey protein mass flow compared to steady flow at steady-state.

The observed improvements in filtration performance with pulsed flow are generally in accordance with our previous lab-scale study [31]. The increased mass flow, induced by enhanced flux and permeation, is the result of improved access to flow shadows causing improved deposit control with less fouling [31]. The small differences in performance improvement with pulsed flow between lab-scale and industrial-scale membranes, particularly regarding whey protein permeation, could arise from the fact that the highest applied axial pressure drop, and thus the flow velocity, was much lower (Δp_L, max_ = 0.83 bar m^−1^) compared to that in the previous study (Δp_L, max_ = 2.55 bar m^−1^) with FSM. The same applies to the amplitude (Δv = 0.26 m s^−1^ versus Δv = 0.60 m s^−1^) [31], as explained above. Frequency and amplitude have both been previously identified to be critical aspects for pulsed flow efficiency [44,45,46,47,54].

#### 3.1.3. Influence of Pulsed Flow on Cleaning Efficiency in Industrial-Scale SWMs

**Figure 6:**
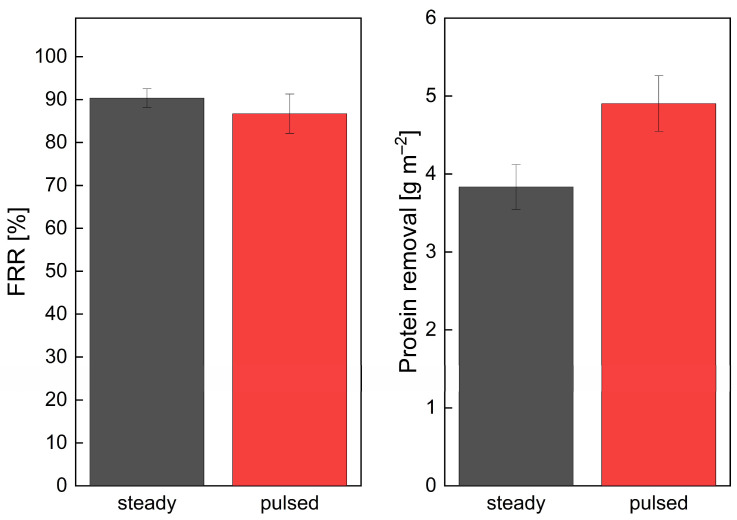
Influence of steady (black) and pulsed (red) flow on FRR (**left**) and protein removal (**right**) during membrane cleaning after skim milk MF. Cleaning conditions: ∆$\dot{V}$ = 10 m^3^ h^−1^ (${\dot{V}}_{\max}$ = 14 m^3^ h^−1^, ${\dot{V}}_{\min}$ = 4 m^3^ h^−1^, $\dot{V}$_avg_ = 9 m^3^ h^−1^) and ∆TMP_cycle_ = 1.00 bar (TMP_max_ = 1.15 bar, TMP_min_ = 0.15 bar, TMP_avg_ = 0.60 bar).

To also examine the effect of pulsed flow on cleaning efficiency with the modified filtration plant for industrial-scale SWMs, cleaning experiments with NaOH at pH 11.3 (c_NaOH_ = 0.03%) were conducted after steady flow filtration. Again, pulsed flow experiments were conducted at the maximum frequency and amplitude settings possible with the current setup. The results of comparing steady and pulsed flow cleaning experiments were evaluated using FRR (Figure 6 left) and total protein removal (Figure 6 right) as assessment criteria.

Regarding FRR, there were no significant differences between flow modes, with 90 ± 2% for steady and 87 ± 5% for pulsed flow. Nonetheless, the protein removal achieved with pulsed flow (4.90 ± 0.36 g m^−2^) was significantly increased by 28% over that achieved with steady flow cleaning (3.83 ± 0.29 g m^−2^). With pulsed flow improving access to flow shadows [29,31,44,51,55,56,57,58,59] and thus improving removal particularly in these areas, the reason for the absence of improvements in FRR could be due to the steady water flux measurements being prone to the same flow shadows behind spacer filaments as steady flow cleaning. As shown in a previous study for FSMs, fouling residues in areas subject to flow shadows could only partially be removed by steady flow cleaning, whereas no distinct residues in those areas could be observed for pulsed flow cleaning [29]. As these observations were only reflected by an increased protein removal but not an increase in FRR, it can be assumed that the additional protein removal near spacer filaments could not be detected by steady flux measurements. This is presumably due to these areas hardly contributing to flux under steady flow, regardless of fouling being present or absent, and translates to an overestimation of hydraulic cleanliness for steady flow and an underestimation thereof for pulsed flow cleaning. This explanation also highlights FRR being insufficient as a singular tool for cleaning evaluation, particularly for membranes subject to flow shadows, such as FSMs or SWMs. Overall, similar to the filtration experiments (Section 3.1.2), the positive results from lab-scale trials could be confirmed, but the benefits were less pronounced, due to reasons explained above.

Another factor when comparing FSM and SWM results is the membrane length, which could also affect the results of pulsation efficiency. Due to the length dependency of Δp_L_, TMP and fouling, the membrane length was previously identified in HFM to affect the cleaning efficiency for flow modes inducing flow reversal [53] but not for conventional steady or pulsed flow [29]. Nonetheless, due to significant geometrical differences between HFMs and SWMs, a declining efficiency of pulsation effects with increasing membrane length in SWMs, e.g., due to propagating flow and pressure waves being partially absorbed by the friction with spacer filaments, cannot be excluded. Nonetheless, the lower flow velocity and amplitude, limited by the maximum pressure drop applicable and thus the stability of SWMs, remain the most probable causes for the observed differences between FSMs and SWMs. Hence, the following sections will investigate an approach to improve module stability in SWMs under operating conditions currently out of reach.

### 3.2. Optimisation of SWMs’ Mechanical Stability by Feed-Side Glue Connections

**Figure 7:**
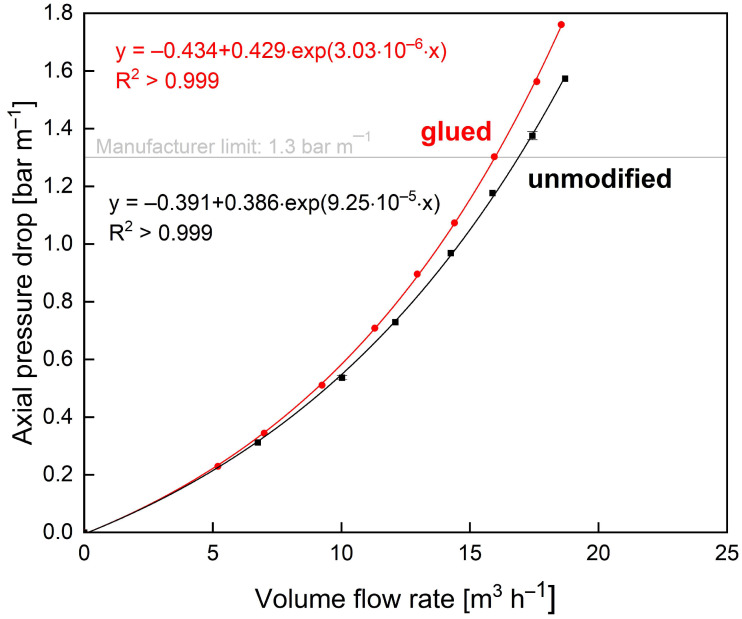
Axial pressure drop at different feed flow rates of the glued (red) and unmodified (black) membrane without an ATD.

First, the effect of glue connections on the filtration behaviour was to be assessed since the glued areas reduce the inlet cross-section of the module. Therefore, the relationship between axial pressure drop and volume flow rates was compared for a glued and an unmodified membrane (Figure 7).

The unmodified membrane reaches the maximum axial pressure drop of 1.3 bar m^−1^ at 16.9 m^3^ h^−1^, and the glued membrane already at 15.9 m^3^ h^−1^. This means that for an identical maximum pressure drop of 1.30 bar m^−1^, the modified membrane could only be operated at a six percent lower volume flow rate than the unmodified membrane. Hence, potential improvements in module stability need to be more pronounced than the enhanced axial pressure drop induced by the glued sections. Otherwise, if glued connections could not provide sufficient stability improvements, the enhanced axial pressure drop would further limit the highest applicable flow velocities. Also, it is to be noted that the glued connections were added to the SWM after its manufacture. Therefore, the areas covered with glue were larger and less well-shaped than they could be when created during the SWM manufacturing process.

**Figure 8:**
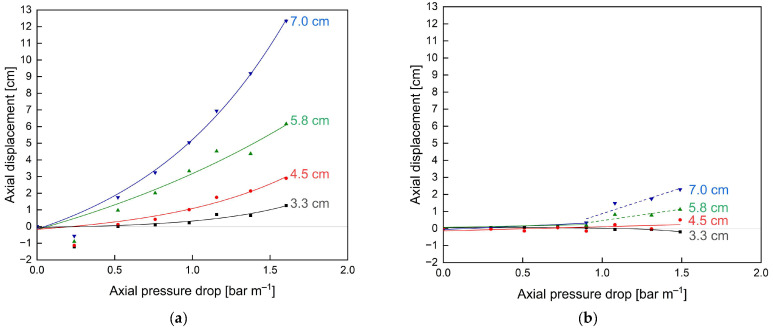
Axial displacement of the unmodified (**a**) and glued (**b**) membrane caused by axial pressure drops at different radial distances to the module centre without an ATD. The grey reference lines indicate no axial displacement. Lines are a guide for the eye.

Furthermore, the effect of additional glue connections on the short-term stability of the membranes without an ATD was assessed in terms of the axial displacement at different radial positions caused by different axial pressure drops (Figure 8).

Due to the absence of an ATD, the critical axial pressure drop, where axial displacement starts to occur, was reached at 0.5 bar m^−1^ for the unmodified membrane (Figure 8a). Beyond this point, the axial displacement increased exponentially as a function of axial pressure drop. Also, the displacement was most pronounced for the membrane parts in the radially outer positions, i.e., farthest away from the central collection tube (7.0 cm), as the pockets are only fixed to the central collection tube and the outer part is only held in place by the friction induced by the module wrapping. Hence, the outer part cannot take up high forces in the axial direction and thus is pushed towards the rear part of the module 879% further than the inner part (12.34 cm displacement at 7.0 cm radial distance versus 1.26 cm displacement at 3.3 cm radial distance) where most of the axial forces can be taken up by the connection to the central collection tube. Even at pressure drops of 1.5 bar m^−1^, above the manufacturer limit of 1.3 bar m^−1^, only a slight axial displacement <1.0 cm of the inner membrane envelope could be observed. Overall, these results emphasise both the instability, particularly of the outer SWM parts, against telescoping as well as the necessity and potential advantages of additional stabilisers, such as glued connections, for module stability.

**Figure 9:**
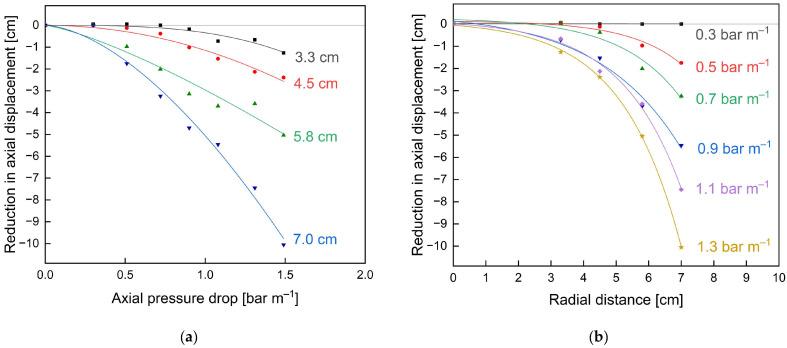
Reductions in axial displacement achieved by the glued membrane compared to the unmodified membrane at different feed flow rates (**a**), i.e., axial pressure drops and different radial distances to the module centre (**b**) without an ATD. The values were obtained by subtracting the displacement of the unmodified membrane from the glued membrane (see Figure 8). Lines are a guide for the eye.

The glued membrane (Figure 8b) showed vastly different results with no displacement up to an axial pressure drop of 1.0 bar m^−1^. This translates to an overall 100% stability increase compared to the unmodified membrane with significant displacements already observed at 0.5 bar m^−1^. Considering the displacements at different radial positions, they are all significantly reduced. For the inner part, i.e., 3.3 cm and 4.5 cm, no significant displacement can be observed for axial pressure drops up to 1.5 bar m^−1^. At 4.5 cm radial distance and 1.5 bar m^−1^, the displacement in the glued membrane was 82% reduced compared to that of the unmodified membrane (0.5 cm versus 2.9 cm). In the outer part (7.0 cm radial distance) at 1.5 bar m^−1^, where displacement was most pronounced for both membranes, the displacement could be reduced by 81% (2.3 cm versus 12.3 cm). An overview of the achieved reductions in axial displacements with the modified membrane compared to the unmodified membrane shows an exponential increase for increasing pressure drops (see Figure 9a) and radial distances to the module centre (see Figure 9b). Hence, improvements of the modified membrane are most pronounced for outer membrane parts and at increased axial pressure drops or flow velocities.

Besides the observed stability increases, axial displacement still occurred due to the increasingly stressed bond joints eventually rupturing at their weakest point. As a consequence, the supportive effect of this bond joint vanished, and axial displacement occurred. Nevertheless, due to the supportive effect of the remaining membrane sheet connections, the resulting axial displacement could be significantly reduced compared to an envelope without glued bond joints. The related failure mechanism was a rupture of the glue connections. An additional failure mechanism, e.g., the axial displacement of the feed spacer, as observed in the used membranes, could not be observed for the glued membranes, which also underlines their enhanced stability.

It is to be noted that due to glue connections being added after SWM manufacturing and for used membranes, the adhesion between the selective and support layer, as well as the glue connections and their geometry, might not be ideal. Hence, it can be assumed that if prepared under ideal conditions, the stability improvements gained by glue connections would be more pronounced.

## 4. Conclusions

This study presented two approaches to successfully reduce the limitations of SWMs regarding cleanability and mechanical stability. The first approach, focusing on processing, transferred and utilised the concept of pulsed flow to industrial-scale membranes by adding a controlled bypass. This led to similar flow characteristics but less distinct process improvements during filtration and cleaning compared to previous lab-scale studies using FSMs [29,31]. While the underlying causes for the observed differences between lab-scale FSMs and industrial-scale SWMs could not entirely be determined, they are presumably due to the reduced flow velocity and pulsation amplitude applicable in SWMs. Nonetheless, significant improvements for both filtration (mass flow +26%) and cleaning performance (protein removal +28%) could be confirmed for pulsed flow. It is to be noted that while this approach was associated with low effort and investment cost on the pilot scale, a transfer to industrial-scale systems, often encompassing several membrane housings, i.e., filtration units, would require an efficient implementation of the additional controlled bypass into each filtration unit. This could be performed by, e.g., combining two filtration units into one pulsation unit where the flow control is managed by a controlled three-way diverting valve instead of a controlled bypass. This way, one filtration unit would be in the high flow rate phase while the other filtration unit would be in the low flow rate phase. One advantage of this approach would be that no pump energy would be left unused as bypass flow but instead split between two filtration units pulsing inversely. Despite the advantages of pulsed flow, the necessity of adding the respective type of pulsation creation to every filtration unit is given for each type of pulsation creation and should thus be considered upon implementation.

The second approach, focusing on membrane construction, applied glue connections between membrane sheets in the radial direction across the membrane width of a used SWM. As a result, the axial pressure drop at a given flow rate was slightly increased. However, the stability against axial displacement without an ATD was increased by ≥100% across the whole membrane diameter. Consequently, the enhanced axial pressure drop at the inlet partly compensated the glued connections’ positive effect. Nevertheless, the positive effect of enhanced stability predominates over the disadvantage of enhanced axial pressure drop. Thus, higher flow rates and increased amplitudes under pulsed flow are expected to be applicable to the glue-connected SWM. Due to glue connections being added under non-ideal conditions, i.e., after manufacturing and for used membranes, it can be assumed that stability improvements would be more pronounced under ideal glueing conditions. In the case of manufacturing the interconnections between the membrane pockets simultaneously with the SWM itself, the bonds could be formed slimmer but longer to leave more free inlet cross-sections. Additionally, the effect of those glue connections might be enhanced by optimising their location, orientation and extent within the membrane module. However, since the effects on module stability could only be assessed with short-term experiments under extreme conditions, i.e., without an ATD, long-term stability tests should be conducted to confirm the enhanced stability for conventional setups with an ATD.

Finally, the combined maximum achievable advantages of using a stabilised SWM with pulsed flow allowing for increased pulsation amplitudes at increased flow rates should be assessed to facilitate weighing the required implementation effort versus the gained advantage, particularly from an economic and ecological point of view.

## Data Availability

The datasets generated during and/or analysed during the current study are available from the corresponding author on reasonable request.
